# From Validation to Assessment of e-Health Literacy: A Study among Higher Education Students in Portugal

**DOI:** 10.3390/healthcare12161626

**Published:** 2024-08-15

**Authors:** Leandro Oliveira, Renata Puppin Zandonadi, Eduardo Yoshio Nakano, Sulaiman Almutairi, Haitham Alzghaibi, Maria João Lima, Edite Teixeira-Lemos, Ariana Saraiva, António Raposo

**Affiliations:** 1 CBIOS (Research Center for Biosciences and Health Technologies), Universidade Lusófona de Humanidades e Tecnologias, Campo Grande 376, 1749-024 Lisboa, Portugal; 2 Polytechnic Institute of Coimbra, Coimbra Health School, Rua 5 de Outubro–S. Martinho do Bispo, Apartado 7006, 3046-854 Coimbra, Portugal; 3 Department of Nutrition, School of Health Sciences, University of Brasilia (UnB), Campus Darcy Ribeiro, Asa Norte, Brasilia 70910-900, Brazil; renatapz@unb.br; 4 Department of Statistics, University of Brasília (UnB), Campus Darcy Ribeiro, Asa Norte, Brasilia 70910-900, Brazil; nakano@unb.br; 5 Department of Health Informatics, College of Applied Medical Sciences, Qassim University, Buraydah 51452, Saudi Arabia; ssmtiery@qu.edu.sa (S.A.); halzghaibi@qu.edu.sa (H.A.); 6 CERNAS Research Centre, Polytechnic University of Viseu, 3504-510 Viseu, Portugal; mjoaolima@esav.ipv.pt (M.J.L.); etlemos3@gmail.com (E.T.-L.); 7 Department of Animal Pathology and Production, Bromatology and Food Technology, Faculty of Veterinary, Universidad de Las Palmas de Gran Canaria, Trasmontaña s/n, 35413 Arucas, Spain; ariana_23@outlook.pt

**Keywords:** e-health, health literacy, higher education, internet, students

## Abstract

Despite their familiarity with technology, higher education students often lack the critical skills needed to assess the credibility of online health information, potentially impacting their health decisions and well-being. This study aims to validate and assess the e-Health Literacy Scale among those in Portuguese higher education. In addition, this study focused on measuring their e-health literacy levels and investigating how these skills relate to different sociodemographic variables. This cross-sectional study was conducted in two phases. Initially, the test–retest reliability and reproducibility of measured e-health literacy were assessed with a convenience sample of 20 participants. Subsequently, the e-health scale was applied to a group of 245 Portuguese higher education students. The research took place from January 2023 to April 2024. The scale exhibited robust internal consistency and reproducibility. Male gender consistently correlates with higher levels of e-health literacy. Students demonstrate good levels of e-health literacy (24/40), reflecting their ability to effectively navigate and utilize health information online. By integrating strategies to further enhance this literacy into university health programs, students can develop essential skills necessary for making informed decisions about their health. This proactive approach not only empowers students to access reliable health resources but also fosters a culture of health literacy that can positively impact their well-being both during their academic journey and beyond graduation.

## 1. Introduction

Health literacy is a comprehensive concept that extends beyond the ability to read medical texts; it involves the capacity to comprehend and effectively apply health information in everyday life [1,2]. In a world where information is widely accessible through the internet, health literacy becomes even more crucial. Individuals with adequate levels of health literacy are more likely to make informed decisions about their health, such as adhering correctly to medical treatments, preventing diseases through healthy habits, and seeking medical assistance when necessary [3,4].

However, despite the proliferation of online health information, many people face significant challenges in accessing, comprehending, and utilizing this information effectively. This is especially true for groups with low health literacy and limited digital skills, who may be disadvantaged when trying to navigate the vast volume of information available on the internet [4,5].

The increasing adoption of information communication technologies (ICTs) in modern society offers new opportunities to promote health literacy [6]. With over half of the global population connected to the internet, ICTs can potentially empower individuals to become informed consumers of health information [7,8]. However, this requires not only internet access but also specific skills to search, critically evaluate, and apply health information found online [4,9].

ICTs play a crucial role in promoting health literacy by facilitating quick and convenient access to health information [5,6]. Through online platforms, individuals can find a variety of resources on medical conditions, treatments, disease prevention, and health promotion [4,10]. However, the quality and reliability of this information can vary widely, underscoring the importance of critical skills to assess the credibility of sources [10,11].

Health e-literacy, a concept encompassing the ability to search, find, comprehend, and evaluate health information online, is essential for navigating this complex digital landscape [5]. The Lily Model, which outlines six essential competencies of health e-literacy, including functional, informational, scientific, and health skills, provides a structured framework for understanding how individuals interact with online health information [11].

As integral members of the digital generation, higher education students are particularly poised to benefit from ICTs in relation to health literacy [9]. Not only do they have frequent internet access, but they are also more likely to utilize digital technologies to seek health information [8,12]. However, studies indicate that even among young adults, there are significant challenges in discerning reliable health information online, which can compromise their health decisions [13,14].

Despite the advantages offered by ICTs in promoting health literacy, there are significant challenges to overcome. One of the primary challenges is the ability to filter accurate and reliable health information from unverified or even misleading sources on the internet [10,13]. Higher education students, while familiar with technology use, may lack the critical skills necessary to assess the quality of online health information, potentially impacting their health and well-being decisions [15].

In addition, disparities in internet access and digital skills among different population groups can perpetuate health inequalities. Vulnerable groups, such as those with low socioeconomic status or older adults, may face additional challenges when trying to access health information online, exacerbating existing disparities in the healthcare system [16].

Research on health literacy among higher education students is crucial for understanding their specific needs and developing effective strategies to enhance their e-health literacy skills [17]. By identifying knowledge and skills gaps, educators and healthcare professionals can design educational programs that strengthen students’ ability to make informed decisions.

Higher education in Portugal is organized into a binary system comprising university education and polytechnic education, offered by both public and private institutions. Private higher education institutions receive prior recognition of public interest from the government. University education includes universities, university institutes, and other university-level establishments. Polytechnic education encompasses polytechnic institutes and other polytechnic-level establishments [18]. In 2022, the number of higher education graduates decreased. The higher education completion rate for individuals aged 25 to 34 was 44.4%, still above the EU average (42%) and close to the EU target (45%). Over the past decade, higher education completion rates increased by 15 percentage points, mainly due to a rise in the number of women completing higher education, which widened the gender gap (from 10.7 to 15.4 percentage points in favor of women). Despite this, more young people are enrolling in higher education. In the 2021/2022 academic year, 433,217 students were enrolled in higher education institutions, setting a new record following the previous high of 411,995 in 2020/2021 [19].

A recent study in Portugal found that 84.1% of students used the internet for more than two hours a day. In the last two months, 42.7% spent less than one hour searching for health information, 25.7% spent two to three hours, and 14.0% spent more than three hours on such searches [20]. Given this extensive use, it is crucial to improve students’ skills in assessing the credibility of online health resources [20]. In fact, studies indicate that higher education students often rely on the internet for health information but may not be adequately prepared to evaluate its credibility [8,21].

The e-Health Literacy Scale (eHEALS) was chosen for our study due to its several strengths: it provides a comprehensive framework for assessing essential e-health skills and has been validated for reliability; consisting of only eight items, it is easy to complete. It has broad applicability across various demographics and health contexts, and its strong theoretical foundation enhances robustness; and, its widespread use in e-health research facilitates comparisons with other studies [22,23,24]. This way, by using eHEALS, we ensure accurate, reliable, and comparable data on e-health literacy.

In today’s digital age, e-health literacy is increasingly crucial, as the ability to effectively find, understand, and use health information online can significantly influence individuals’ health outcomes. Higher education students, who frequently rely on the internet for information, represent a pivotal demographic for assessing and enhancing e-health literacy. To our knowledge, no studies in Portugal evaluated the properties of eHEALS within this specific group, only in adolescents [22,23]. By validating the eHEALS [24] in this population, the study seeks to ensure the reliability and applicability of the tool, thereby contributing to the development of more effective educational strategies and interventions aimed at improving health literacy and outcomes in this critical group.

Investing in health literacy education during their academic years can significantly impact individuals’ future ability to manage their own health and that of those around them [25]. Therefore, this study aims to validate and assess eHEALS among Portuguese higher education students.

## 2. Materials and Methods

This study was conducted in two steps: test–retest and reproducibility of e-health, and the application of e-health among Portuguese higher education students. 

The research took place between January 2023 and April 2024. The study was conducted in accordance with the Declaration of Helsinki and approved by the Ethics Committee of the School of Health Sciences and Technologies of Lusófona University (P10-22, 7 December 2022). 

### 2.1. Test–Retest and Reproducibility of e-Health

The reproducibility of the e-health literacy assessment was analyzed before a more extensive application. It was performed with test–retest reliability. For this purpose, we invited 20 individuals (convenience sample) to evaluate the eHealth questionnaire’s reproducibility (test–retest agreement). The reproducibility was performed using the responses of these 20 Portuguese adult individuals who answered the questionnaire twice at different times with an approximate interval of 1 week between answers (minimum of 48 h and maximum of 15 days). The reproducibility of the e-health assessment was verified through the Intraclass Correlation Coefficient (ICC) with two random effect models and absolute agreement definition. The analysis was based on a mean measure, and values equal to or greater than 0.7 indicate the instrument has good reproducibility [26,27].

### 2.2. e-Health Application among Portuguese Higher Education Students

To validate and evaluate e-health among a sample of higher education students, we used an instrument composed of two parts: (i) sociodemographic and health-related questions; (ii) eHEALS [24]—Appendix A. 

The study targets undergraduate (bachelor’s) and graduate (master’s and PhD) students. Additionally, participants were asked if they were enrolled in postgraduate programs or other courses.

eHEALS was created by Norman and Skinner [24]. It includes eight single-dimensional items rated on a 5-point Likert scale, where (1) indicates complete disagreement and (5) indicates complete agreement. The final score ranges from 8 to 40, with higher scores indicating a greater ability of the individual to obtain reliable information on health-related topics from the internet [24]. In this study, the scale translated into Portuguese by Tomás et al. [23] was used, originally applied in adolescents.

The process of validating a questionnaire requires 20 respondents per item (20:1) [28]. Therefore, it was estimated that the minimum sample size of 160 participants would be necessary to validate the e-health questionnaire composed of 8 items (the questionnaire contains 10 items, but only 8 are used in the score since they are part of the health scale).

The instrument was applied using Google Forms to a convenience sample of higher education students. Participants were recruited from Portuguese higher education institutions (private and public) from north to south, including the islands.

An email requesting the dissemination of the questionnaire to their students was sent to at least one higher education institution in each NUTS II region of Portugal: North, Center, Lisbon Metropolitan Area, Alentejo, Algarve, Autonomous Region of the Azores, and Autonomous Region of Madeira. The students were then encouraged to share the questionnaire with their peers using the snowball method, spreading the link through social media, email, and messaging apps. The data collection period occurred between January and April 2024. The first page of the survey on the platform presented the informed consent form, detailing the aim of the study and the inclusion criteria: (i) being 18 years old or older; (ii) being a higher education student in Portugal. Individuals who did not agree to participate were directed to a page thanking them for their time, while those who agreed were directed to the first page of the questionnaire, which included sociodemographic and health-related questions, followed by the e-Health Literacy Scale.

Sociodemographic variables included: gender; age; place of residence; education level; area of study; and type of higher education institution (public—state; public—non-state; private). The health-related variables were as follows: height (meters—m) and weight (kilograms—kg) (self-reported); and previous diagnosis of chronic diseases with current medication (self-reported).

The responsiveness of e-health was verified by the floor and ceiling effects. The floor effect is observed when e-health produces a score equal to 8 (minimum value). The ceiling effect occurs when the eHealth reaches maximum value (score equal to 40). Internal consistency was evaluated using Cronbach’s alpha, and a value equal to or greater than 0.7 indicates good internal consistency [27].

The scores of the e-health assessment were described in terms of means and standard deviations (SD). Analysis of Variance (ANOVA) followed by Tukey’s post-hoc tests and non-paired Student’s *t*-test were used to compare the scores for e-health with the variables of interest. All tests were performed considering two-tailed hypotheses and a 5% significance level. The analyses were performed using IBM SPSS (version 26, IBM SPSS Statistics for Windows, IBM Corp, Armonk, NY, USA).

## 3. Results

Test–retest analysis indicated that the questionnaire presented good reproducibility (ICC = 0.957) (Table 1).

After confirming the reproducibility of the e-health questionnaire, it was administered to a larger sample (Table 2). From a total of 308 students who agreed to participate in the study, only 245 completed the questionnaire correctly. These are the participants included in the study. The majority of participants were undergraduate students (83.3%), women (77.1%), aged up to 24 years old (80%), residing in the metropolitan area of Lisbon (63.7%), with no diagnosed chronic diseases (80.8%), and of normal weight (72.2%).

Table 3 shows data from the internal consistency evaluation. Analyses revealed Cronbach’s alpha coefficients = 0.850 for e-health scale, representing good internal consistency (≥0.7). The e-health literacy assessment showed good acceptability, showing total floor and ceiling effects ≤ 3.7% [29].

Table 4 presents scores of the e-health scale subcategorized by sociodemographic variables and health characteristics. The e-health only presented significant differences in gender. The results reveal that men have significantly higher e-health literacy scores than women. No significant differences were observed based on age, region, level of course, professional situation, chronic disease status, or health appointment attendance. There is a trend suggesting lower e-health literacy scores with higher BMI, but this difference is not statistically significant.

Tables S1 and S2 show the frequency of e-health responses per item (Table S1) and the frequency of chronic diseases reported by students.

## 4. Discussion

This study aimed to validate eHEALS for Portuguese higher education students and to ensure the tool’s reliability and applicability. The objective was to assess the scale’s effectiveness in measuring e-health literacy within this specific population and to confirm that it consistently provides accurate and relevant results. By doing so, the study sought to establish the scale as a dependable instrument for evaluating students’ ability to access, understand, and utilize online health information. This validation process was crucial for enhancing the tool’s credibility and ensuring its practical use in educational and research settings.

eHEALS demonstrates strong internal consistency, with all items contributing effectively to the assessment of e-health literacy in the Portuguese higher education students population. Comparisons with the validation values of the original scale demonstrated consistent internal consistency [24], mirroring findings from studies conducted among Portuguese students attending secondary education [22,23].

The e-health literacy values observed in our sample (mean = 25.05, SD = 6.08) suggest that this sample of Portuguese higher education students exhibit robust levels of competence in navigating digital health information. This finding aligns with similar studies conducted in diverse settings [20,30,31,32], reinforcing the notion of generally positive eHealth literacy levels among higher education students.

Similar to our study, several others [24,33,34,35,36] have reported that being male consistently correlates with higher levels of eHealth literacy. In contrast, one study [37] found that women were associated with higher e-health literacy. These findings underscore a gender disparity in e-health literacy across various research contexts, emphasizing the necessity for further exploration into the underlying factors contributing to these differences. Factors such as access to information, digital literacy skills, and attitudes towards health information-seeking are likely influential in shaping e-health literacy outcomes among different genders [38].

Age does not appear to be a decisive factor in determining e-health literacy levels, as supported by the findings of Norman and Skinner [24]. Nonetheless, other research consistently links younger age with higher e-health literacy levels [34,39,40,41]. This suggests that while age-related variations in e-health literacy are observed in some studies, such associations were not evident in our study of Portuguese higher education students. This discrepancy might be due to factors such as differences in technology adoption, access to health information, and individual health information-seeking behaviors, which can influence e-health literacy outcomes across different studies. In fact, a study involving 288 participants aged 18 to 65 from Portugal revealed that all participants (100%) had sought health information online. However, the majority of respondents (66.0%) did not discuss this information with their healthcare professionals. Additionally, there was a demand for more information on ethical medications, with 80.6% of participants seeking further details and 74.1% finding advertising formats useful. Google was the primary search engine used (98.2%), though other resources, like the Infarmed website, were also accessed [42]. In a recent study involving higher education students in Portugal, it was observed that students with higher levels of e-health literacy tended to use a wider range of health information sources, including official websites and sites managed by health professionals. Conversely, students with lower e-health literacy levels were more likely to rely on social networks, such as Google, for health-related information [20].

Higher education has consistently been shown to be associated with higher levels of e-health literacy [33,36,37,38,39,40,41,42]. Moreover, research focusing specifically on college students has revealed that higher academic levels (e.g., year 3 compared to year 1) and the specific choice of major significantly influence e-health literacy [43,44]. These studies underscore the critical role of education in shaping individuals’ ability to navigate and utilize health information effectively in digital environments. Contrary to our study, findings from these studies did not reveal significant differences between educational qualifications, particularly between bachelor’s and master’s or PhD degrees, in terms of e-health literacy levels. This suggests that while higher education generally enhances e-health literacy, the specific level of academic attainment within the higher education spectrum may not consistently influence proficiency in navigating digital health information [20].

Our study found no significant differences in e-health literacy based on whether individuals had a chronic disease, attended health appointments, or had a specific body mass index. However, other studies have indicated relationships between e-health literacy and health-promoting behavior, particularly in areas such as exercise [30,36,43,44,45], healthy eating [36,44,45], sleep, abstaining from harmful substances, and maintaining sexual health [44]. These findings underscore the broad impact of eHealth literacy on promoting healthy behavior across different populations and settings.

Despite the present study focusing on higher education students, it is important to remember that integrating e-health literacy education in primary and secondary education establishes foundational skills that are further developed in higher education. In primary education, the focus is on basic skills such as understanding and recognizing health information and digital literacy. In secondary education, students learn to evaluate the credibility of online health information and understand privacy issues. Curriculum integration should be age-appropriate, with interactive methods in primary education and practical activities in secondary education. In higher education, e-health literacy education becomes more advanced, focusing on research and critical analysis. This continuity ensures a comprehensive education and prepares students to critically engage with health information throughout their lives.

Additionally, further studies should be conducted at different educational levels to determine if there are differences in the use of the e-health literacy scale, ensuring the effectiveness and appropriateness of methodologies applied to each age group.

While our study contributes valuable insights into e-health literacy among Portuguese higher education students, further research is needed to elucidate the complex determinants and implications of digital health literacy, including addressing gender disparities and understanding age-related dynamics. Effective leveraging of educational strategies is crucial to enhancing e-health literacy and promoting equitable access to health information in digital environments. Advancing e-health literacy not only enhances individual health literacy skills but also contributes to broader public health goals. By promoting a culture of informed health-related decision making and leveraging digital tools responsibly, we can foster healthier communities and ultimately achieve more equitable health outcomes for all.

### Strengths and Limitations

The study followed a rigorous methodology with two main phases: test–retest and application among higher education students. This structured approach provided a solid foundation for analyzing the reproducibility and validity of the e-health questionnaire. Despite using a convenience sample obtained by the snowball method, the inclusion of students from various higher education institutions across Portugal, encompassing different regions and the islands, contributed to a relatively diverse sample. This diversity offered a broad perspective on eHealth literacy among students. Focusing on e-health literacy is highly relevant in the current context, where digital health information access and use are crucial for making informed decisions.

However, the use of convenience sampling in both phases of the study may introduce selection bias, limiting the generalizability of the results. Although the sample size was statistically adequate for validation purposes, the sample’s composition might still skew results [46]. Certain demographics or institutions could be over-represented, which may not provide a fully balanced view of e-health literacy across all student groups. Additionally, the study relied on self-reported data, which poses its own set of challenges. Respondents may have provided inaccurate information, intentionally or unintentionally, or may have answered in a way they believe is socially desirable rather than truthfully. Such biases can affect the reliability of the data and the overall findings. The psychometric properties of the e-health questionnaire were validated within this specific context. To ensure the instrument’s broader applicability and reliability, further validation is needed across diverse populations and settings. Addressing these limitations is crucial for refining the questionnaire and enhancing its accuracy and generalizability.

## 5. Conclusions

eHEALS has proven to be a reliable and valid tool for assessing e-health literacy among Portuguese higher education students. The high overall levels of e-health literacy suggest that this population is relatively adept at navigating and utilizing online health information. However, significant variations, particularly with men scoring higher than women on the e-health literacy scale, highlight the necessity for targeted educational interventions. To address these disparities, we recommend the development of specific educational strategies, such as gender-sensitive workshops and tailored digital literacy programs, which could further enhance e-health literacy. These strategies should focus on addressing the unique needs and challenges faced by different groups, thereby improving e-health literacy comprehensively across university students in Portugal.

## Figures and Tables

**Table 1:** Test–retest agreement of e-health score (n = 20).

Item	Test Mean (SD)	Retest Mean (SD)	ICC * 95% CI
Score	30.25 (4.78)	29.40 (5.91)	0.957 (0.886; 0.983)

* Intraclass Correlation Coefficient.

**Table 2:** Sociodemographic characteristics of the individuals (n = 245).

		Sample (n = 245)	
		Frequency	%
Gender	Women	189	77.1%
	Men	56	22.9%
Age	Up to 19 years	86	35.1%
	20 to 24 years	110	44.9%
	25 years or older	49	20.0%
Region	Center	63	25.7%
	Lisbon metropolitan area	156	63.7%
	Other	26	10.6%
Level of the course you are attending	Bachelor’s degree	204	83.3%
	Master or PhD degree	41	16.7%
Professional situation	Student	222	90.6%
	Student worker	23	9.4%
Do you have any chronic disease?	No	198	80.8%
	Yes	47	19.2%
Are you being followed up in health appointments?	No	102	41.6%
	Yes	143	58.4%
BMI	Up to 18.99 kg/m^2^	23	9.4%
	19 to 24.99 kg/m^2^	177	72.2%
	25 to 29.99 kg/m^2^	32	13.1%
	30 kg/m^2^ or more	13	5.3%

Other: Norte, Alentejo, Algarve, Autonomous Region of Madeira.

**Table 3:** e-Health score, responsiveness, and internal consistency of the questionnaire (n = 245).

	Mean (SD)	Median (IQR *)	Range	Floor Effect (%)	Ceiling Effect (%)	Cronbach’s Alpha
e-Health	25.05 (6.08)	26 (22–30)	8–32	3.7%	0%	0.850

* IQR: Interquartile range.

**Table 4:** Scores of the e-health scale subcategorized by sociodemographic variables and health characteristics (n = 245).

Sex *	**Men**	**Women**			***p***
Mean (SD)	25.72 (5.68)	22.79 (6.85)			0.001
Age (years) **	**Up to 19**	**20 to 24**	**25 or more**		***p***
Mean (SD)	24.99 (4.75)	25.24 (6.37)	24.76 (7.46)		0.893
Region **	**Center**	**Lisbon metropolitan area**	**Other**		***p***
Mean (SD)	24.98 (6.38)	25.07 (5.89)	25.12 (6.71)		0.994
Level of the course you are attending *	**Bachelor’s degree**	**Master’s or PhD degree**			***p***
Mean (SD)	25.32 (5.79)	23.73 (7.32)			0.197
**Professional situation**	**Student**	**Student worker**			***p***
	25.23 (6.09)	23.30 (5.82)			0.148
**Do you have any chronic disease?**	**No**	**Yes**			***p***
Mean (SD)	25.16 (5.89)	24.60 (6.89)			0.567
**Are you being followed up in health appointments?**	**No**	**Yes**			***p***
Mean (SD)	24.73 (6.57)	25.29 (5.72)			0.478
BMI kg/m^2^ **	**Up to 18.99**	**18.5 to 24.99**	**25 to 29.99**	**30 or more**	***p***
Mean (SD)	24.09 (5.55)	25.53 (5.92)	24.63 (6.04)	21.38 (8.22)	0.087

* Student *t*-test. ** Anova with Tukey post-hoc test. Other: Norte, Alentejo, Algarve, Autonomous Region of Madeira.

## Data Availability

Data are contained within the article.

## Supplementary Materials

The following supporting information can be downloaded at: https://www.mdpi.com/article/10.3390/healthcare12161626/s1, Table S1: Frequency of e-health responses per item (n = 245).; Table S2: Frequency of diseases reported by students (n = 245).

## Appendix A

**Table A1:** e-Health Literacy scale.

Portuguese	English (Free Translation)	1. Absolutamente Nada Importante/ 1. Absolutely Not Important	2. Pouco Importante/ 2. Not Very Important	3. Neutro/ 3. Neutral	4. Importante/ 4. Important	5. Muito Importante/ 5. Very Important
Questões Acessórias	Additional Questions					
Até que ponto considera que a internet é útil para o/a ajudar a tomar decisões sobre a sua saúde?	To what extent do you consider the internet to be useful in helping you make decisions about your health?					
Até que ponto considera importante para si poder ter acesso a recursos sobre saúde na internet?	How important do you consider it to be to have access to health resources on the internet?					
Escala de e-Literacia em Saúde	e-Health Literacy Scale	1. Discordo totalmente/ 1. Strongly disagree	2. Discordo/ 2. Disagree	3. Indeciso/ 3. Undecided	4. Concordo/ 4. Agree	5. Concordo totalmente/ 5. Strongly agree
1. Sei quais são os recursos sobre saúde disponíveis na internet.	1. I know what health resources are available on the internet.					
2. Sei onde encontrar recursos úteis sobre saúde na internet.	2. I know where to find useful health resources on the internet.					
3. Sei como encontrar recursos úteis sobre saúde na internet.	3. I know how to find useful health resources on the internet.					
4. Sei como usar a internet para responder às minhas perguntas sobre saúde.	4. I know how to use the internet to answer my health questions.					
5. Sei como usar a informação sobre saúde que encontro na internet para me ajudar.	5. I know how to use the health information I find online to help me.					
6. Consigo avaliar os recursos sobre saúde que encontro na internet.	6. I can evaluate the health resources I find on the internet.					
7. Sei distinguir os recursos de elevada qualidade dos de fraca qualidade entre os recursos sobre saúde da internet.	7. I can distinguish high-quality resources from low-quality resources among health resources on the internet.					
8. Sinto-me confiante a usar informação da internet para tomar decisões sobre saúde.	8. I feel confident using information from the internet to make health decisions.					
