# Supplementary material for: From Validation to Assessment of e-Health Literacy: A Study among Higher Education Students in Portugal
Source: Healthcare (Basel). 2024 Aug 15;12(16):1626. doi: 10.3390/healthcare12161626 (PMC11353653; doi:10.3390/healthcare12161626)
Supplement: Supplementary file 1 [file healthcare-12-01626-s001.zip › healthcare-3101961-supplementary.pdf]

Table S1: Frequency of e-health responses per item (n=245).

Item	Likert Scale*				
	1	2	3	4	5
Q3	27 (11.0%)	18 (7.3%)	70 (28.6%)	130 (53.1%)	0 (0%)
Q4	43 (17.6%)	23 (9.4%)	42 (17.1%)	137 (55.9%)	0 (0%)
Q5	42 (17.1%)	19 (7.8%)	51 (20.8%)	133 (54.3%)	0 (0%)
Q6	36 (14.7%)	20 (8.2%)	52 (21.2%)	137 (55.9%)	0 (0%)
Q7	37 (15.1%)	17 (6.9%)	43 (17.6%)	148 (60.4%)	0 (0%)
Q8	33 (13.5%)	15 (6.1%)	68 (27.8%)	129 (52.7%)	0 (0%)
Q9	47 (19.2%)	12 (4.9%)	55 (22.4%)	131 (53.5%)	0 (0%)
Q10	29 (11.8%)	51 (20.8%)	89 (36.3%)	76 (31.0%)	0 (0%)

Q3: I know what health resources are available on the internet.
Q4: I know where to find useful health resources on the internet.
Q5: I know how to find useful health resources on the internet.
Q6: I know how to use the internet to answer my health questions.
Q7: I know how to use the health information I find on the internet to help me.
Q8: I can evaluate the health resources I find on the internet.
Q9: I can distinguish between high-quality and low-quality health resources on the internet.
Q10: I feel confident using information from the internet to make health decisions.
* Totally disagree (1) to totally agree (5).

Table S2: Frequency of diseases reported by students (n=245).

Disease*	Frequency	%
Respiratory diseases	20	8.2%
Food allergy	10	4.1%
Lactose intolerance	10	4.1%
Vision problems	9	3.7%
Stress, depression, anxiety	9	3.7%
Anemia	6	2.4%
Overweight/obesity	4	1.6%
Gastrointestinal diseases	4	1.6%
Cardiovascular diseases	3	1.2%
Endometriosis	3	1.2%
High blood pressure	3	1.2%
Thyroid diseases	3	1.2%
Other diseases/problems of the nervous system	3	1.2%
Diabetes	2	0.8%
Bone-joint diseases	2	0.8%
Celiac disease	1	0.4%
Rheumatic diseases	1	0.4%
Severe atopic eczema	1	0.4%
Psioriasis	1	0.4%
Dyslipidemia	1	0.4%
Insomnia	1	0.4%
Psychiatric	1	0.4%
Kidney diseases	1	0.4%

* A student can present more than one disease.
